# Relapse of *NPM1*-Mutated AML with Extramedullary Manifestation 17 Years after Allogeneic Hematopoietic Stem Cell Transplantation

**DOI:** 10.1155/2022/3317936

**Published:** 2022-12-28

**Authors:** Jan Braune, Kathrin Rieger, Olga Blau, Ulrich Keller, Lars Bullinger, Jan Krönke

**Affiliations:** ^1^Department of Hematology, Oncology and Cancer Immunology, Charité-Universitätsmedizin Berlin, Freie Universität Berlin and Humboldt-Universität zu Berlin, Berlin, Germany; ^2^German Cancer Consortium (DKTK) Partner Site Berlin and German Cancer Research Center (DKFZ), Heidelberg, Germany; ^3^Berlin Institute of Health (BIH), Berlin, Germany

## Abstract

The majority of patients with acute myeloid leukemia (AML) with the *NPM1* mutation achieve remission with intensive chemotherapy. However, many patients subsequently relapse, which occurs frequently within the first 2–3 years after therapy, while late relapse after more than 10 years is rare and can also represent secondary/therapy-associated AML without the NPM1 mutation. Here, we present a case of *NPM1*-mutated AML that developed medullary and extramedullary relapse 17 years after allogeneic stem cell transplantation, maintaining the *NPM1* mutation and all other genetic alterations detected at first diagnosis. This exceptionally long latency between diagnosis and relapse of a genetically highly related leukemic clone implies the existence of therapy-resistant, persisting dormant leukemic stem cells in *NPM1* mutant AML.

## 1. Introduction

*NPM1* mutations are among the most frequent genetic alterations in acute myeloid leukemia (AML) and are associated with distinct morphological, genetic, and clinical features. Intensive chemotherapy with or without allogeneic stem cell transplantation (allo-HSCT) leads to remission in 70–90% of patients with *NPM1*^mut^ AML, but about 30–50% of patients relapse due to persistent leukemic stem cells [1]. Most AML relapses occur early, usually within 12–18 months after the first complete remission (CR1), while late (LR) and very late relapses (VLR), defined as >3 years and >5 years after CR1, respectively, are rare and occur in 1–3% of the patients [2, 3], with only a few case studies describing relapses after more than 10 years [2, 4–7]. Recent studies revealed that many very late relapses are secondary, therapy-related AMLs with a distinct genetic profile as compared to the first AML at diagnosis [8, 9]. Extramedullary (EM) AML is a rare manifestation of AML and occurs in only 2–9% of newly diagnosed patients [10]. Nevertheless, it occurs more frequently in relapsed patients, and EM relapses have been reported more frequently in transplant recipients than in patients treated with chemotherapy alone.

**Figure 1:**
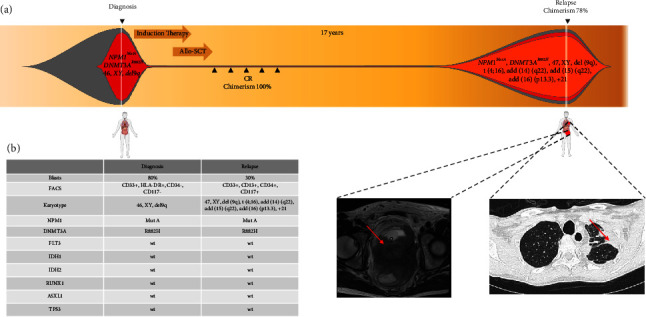
(a) Schematic presentation of treatment and genetic evolution in a patient with *NPM1*-mutated AML. The patient had no evidence of extramedullary disease at diagnosis and presented with a chloroma in the lesser pelvis and pulmonary lesions at relapse, as shown in the MRI and CT scan, respectively. (b) AML characteristics of leukemia at diagnosis and relapse.

Here, we report the case of a patient with *NPM1*-mutated AML who developed a bone marrow (BM) and EM relapse 17 years after the first diagnosis (Figure 1).

## 2. Case Presentation

A 23-year-old male patient was diagnosed with AML (initial white blood cell count: 50/nL; hemoglobin: 3.6 g/dL; platelet count: 67 × 10^3^/*μ*L) in 2003. A BM biopsy showed 80% blasts with monocytic features without evidence of extramedullary disease. Conventional cytogenetics revealed a 46, XY, del(9q) karyotype. Flow cytometry revealed the expression of CD33 and HLA-DR, without CD34 or CD117 expression. Molecular genetics and MRD assessment were initially not performed as they were not available at that time. No relevant comorbidities, no family history of malignancies, and no known environmental exposures to substances that damage DNA were reported. The patient received intensive induction therapy with cytarabine, daunorubicin, and thioguanine and achieved a complete remission. He then underwent allogeneic HSCT from an HLA-identical sibling, which was well tolerated without the development of GvHD. Bone marrow assessment revealed a CR with complete donor chimerism one month after transplantation until the end of follow-up seven years after transplantation.

In 2020, 17 years after transplantation, the patient was admitted to the hospital suffering from severe renal impairment, urine retention, and a mechanical ileus. MRI of the abdomen revealed a 12 × 9 cm large mass infiltrating the lesser pelvis. Furthermore, a computed tomography (CT) scan of the chest revealed lesions in both lungs suspicious of metastases. However, histologic analysis of the tumor that infiltrated the colon mucous membrane showed myeloid leukemic cells that were strongly positive for CD33 and CD117 and negative for CD34.

Next, a BM biopsy was taken, which revealed a 30% infiltration of the BM by myeloid leukemia blasts with monocytic differentiation positive for CD13, CD33, CD34, and CD117. By molecular diagnostics, we detected an *NPM1*-Type A mutation and a *DNMT3A* R882H mutation, while no mutations were found in *FLT3*, *IDH1/IDH2*, *RUNX1*, *TP53*, or *ASXL1*. Cytogenetics revealed the same del(9) (q22q34) as 17 years before, in addition to further chromosomal aberrations in the same metaphases 47, XY, t(4; 6) (p16; p21), del(9) (q22q34)x2, add(14) (q22), add(15) (q22), add(16) (p13.3),and +21.

Retrospective analyses of the diagnostic AML sample obtained 17 years before revealed that the *DNMT3A* R882H and *NPM1* Type A mutations were already present at initial diagnosis, while no other mutations were found in *FLT3*, *IDH1/IDH2*, *RUNX1*, *TP53*, or *ASXL1*. A drop in donor chimerism revealed that leukemia originated from the host and not the donor. The persistence of two identical gene mutations and one chromosomal aberration highly implies that relapse evolved directly from persisting cells of the initial leukemic clone rather than an independent clone with newly acquired aberrations.

After induction therapy with cytarabine and daunorubicin, followed by consolidation therapy with intermediate-dose cytarabine and radiation of the pelvic chloroma, a second complete hematologic remission was achieved with complete regression of the pelvic tumor. Radiotherapy of the lung and pelvis was performed. A second HSCT from an HLA haploid donor was planned, but unfortunately, the patient died due to neutropenic sepsis during consolidation therapy.

## 3. Discussion

This case represents one of the latest relapses described in AML with a confirmed *NPM1* mutation at both diagnosis and relapse. The presence of the same gene mutations, *NPM1*^mutA^ and *DNMT3A*^R882H^, as well as a del(9q), strongly argues that the second AML directly evolved from the initial AML clone [8]. While a complex karyotype that was detected in the relapsed AML in addition to the initial alterations is frequently observed in patients exposed to DNA damaging agents, typical therapy-associated aberrations such as deletions 5 or 7 or mutations in *TP53* [11] were absent. This is in contrast to our previous findings that very late reoccurrence of leukemia in *NPM1*^mut^ AML often represents a genetically distinct, therapy-associated AML without *NPM1* mutation [8, 9].

Bertoli et al. showed that “true,” genetically stable, very late relapses after 5 years are more common in patients with the *NPM1* mutation than in AML without the *NPM1* mutation [3]. Watts et al. recently described four cases with very late relapse [12] that were similar to our patient: they had a monocytic phenotype, underwent allo-HSCT, and relapsed with the extramedullary disease. While molecular diagnostic data was not available for any of these cases, monocytic differentiation and extramedullary disease are indicative that these cases harbored an *NPM1* mutation. Bolli et al. described a case of a myeloid sarcoma with an *NPM1* mutation occurring 20 years after the first diagnosis of AML but no genetic information was available at the initial diagnosis [5]. EM disease, although a rare manifestation of AML, is more frequently seen at relapse and after allo-HSCT compared to standard chemotherapy, suggesting a differing underlying pathogenic mechanism [10, 12]. Characteristics that seem to correlate with EM relapse are VLR, monocytic differentiation, favorable cytogenetics, younger age, and *NPM1* mutations [3, 10, 12], all of which were present in our case.

The underlying pathophysiologic mechanisms of such long quiescence of a leukemic stem cell are unclear. The microenvironment has a strong influence on normal and leukemic stem cells, and the transition of such a cell outside the bone marrow may have induced an awakening from dormancy. Decreased immunosurveillance may be a possible factor. Graft versus leukemia might not be that effective outside the BM, explaining the higher rates of EM relapse after allo-HSCT [13–15].

Very late relapse AML with clear proof of a genetic relationship, as in our case, has several implications. From a clinical perspective, the occurrence of very late hematologic and extramedullary relapse in *NPM1*^mut^ AML has to be considered and warrants awareness of the possibility of EM relapse. AML cases with VLR further demonstrate that leukemia stem cells (LSCs) may persist for many years, most likely in a dormant state that is invisible to the immune system, even after allo-HSCT. It is conceivable that many patients in long-term remission also have persisting, dormant LSCs, as also suggested by the detection of low MRD levels in patients in long-term remission [16]. The reasons for the “awakening” of persisting LSCs are not clear and warrant the further molecular and functional characterization of dormant LSCs. Furthermore, understanding how AML cells persist in therapy over many years may help find ways to eradicate them and cure patients.

## Data Availability

The data used to support the findings of this study are available from the corresponding author upon request.
